# Microsomal prostaglandin E synthase-1 gene deletion impairs neuro-immune circuitry of the cholinergic anti-inflammatory pathway in endotoxaemic mouse spleen

**DOI:** 10.1371/journal.pone.0193210

**Published:** 2018-02-22

**Authors:** Priya Revathikumar, Johanna Estelius, Utsa Karmakar, Erwan Le Maître, Marina Korotkova, Per-Johan Jakobsson, Jon Lampa

**Affiliations:** Rheumatology Unit, Department of Medicine, Solna, Karolinska Institutet, Karolinska University Hospital, Stockholm, Sweden; University of California Los Angeles, UNITED STATES

## Abstract

The cholinergic anti-inflammatory pathway (CAP) is an innate neural reflex where parasympathetic and sympathetic nerves work jointly to control inflammation. Activation of CAP by vagus nerve stimulation (VNS) has paved way for novel therapeutic strategies in treating inflammatory diseases. Recently, we discovered that VNS mediated splenic acetylcholine (ACh) release and subsequent immunosuppression in response to LPS associated inflammation is impaired in mice lacking microsomal prostaglandin E synthase-1 (mPGES-1) expression, a key enzyme responsible for prostaglandin E2 synthesis. Here, we have further investigated the consequences of mPGES-1 deficiency on various molecular/cellular events in the spleen which is critical for the optimal functioning of VNS in endotoxaemic mice. First, VNS induced splenic norepinephrine (NE) release in both mPGES-1 (+/+) and (-/-) mice. Compared to mPGES-1 (+/+), immunomodulatory effects of NE on cytokines were strongly compromised in mPGES-1 (-/-) splenocytes. Interestingly, while LPS increased choline acetyltransferase (ChAT) protein level in mPGES-1 (+/+) splenocytes, it failed to exert similar effects in mPGES-1 (-/-) splenocytes despite unaltered β_2_ AR protein expression. In addition, nicotine inhibited TNFα release by LPS activated mPGES-1 (+/+) splenocytes *in vitro*. However, such immunosuppressive effects of nicotine were reversed both in mPGES-1 (-/-) mouse splenocytes and human PBMC treated with mPGES-1 inhibitor. In summary, our data implicate PGE2 as an important mediator of ACh synthesis and noradrenergic/cholinergic molecular events in the spleen that constitute a crucial part of the CAP immune regulation. Our results suggest a possible link between cholinergic and PG system of CAP that may be of clinical significance in VNS treatment.

## Introduction

The discovery of cholinergic anti-inflammatory pathway (CAP), made a few decades ago, has challenged our understanding of the autonomy of our immune system in many ways including the ability of cytokines to activate nerves and the capacity of immune cells to secrete neurotransmitters. CAP is a combination of both parasympathetic (vagus nerve) and sympathetic (splenic nerve) nervous systems that works hand in hand to not only recognize homeostatic imbalances induced by inflammatory cytokines but also respond in a controlled, timely and localized manner to limit inflammation and restore homeostasis. Onset of peripheral inflammation, caused by the invasion of foreign agents or pathogens, is known to be orchestrated by the amplified production of pro-inflammatory cytokines such as TNFα, IL-1β and IL-8 by activated innate immune cells. Such immune responses eventually result in the elimination of the causative agent and what follows is an active resolution phase and restoration of homeostasis. Any setback in resolution might result in exacerbated inflammation and occurrence of chronic inflammatory conditions [1].

Resolution can be achieved by production of anti-inflammatory cytokines, biochemical mediators, resolving lipid mediators and generation of regulatory immune cells such as M2 macrophages [2, 3]. In addition, the autonomic nervous system also has a substantial role in limiting peripheral inflammation through reflexes, where CAP is an important part of the efferent arm, however, the exact mechanism leading to norepinephrine (NE) release in the spleen is still incompletely understood and alternative mechanisms have been suggested [4, 5]. Using animal sepsis models, Tracey and colleagues have demonstrated the main cellular and molecular events linking the immune and nervous systems in the following sequence. Afferent vagal nerves sense and transfer the inflammatory signals from the periphery to the brainstem of the CNS. These incoming signals are further processed in the CNS and action potentials are sent through the efferent vagal nerves which in turn lead to NE release in the spleen. It was suggested that the sympathetic splenic nerve was activated by vagal signaling via post synaptic α7 nicotinic acetylcholine receptors (α7nAChR) in the celiac-mesenteric ganglion [6], however this connection is now up for debate, and expression of α7nACh receptors on immune cells are also shown to be integral for normal functioning of the CAP [7]. Stimulation of the splenic nerve results in NE release and activation of β_2_ adrenergic receptors (AR) on CD4^+^ CD44^hi^ CD62L^lo^ effector memory T lymphocytes residing in close proximity to nerve endings [8, 9]. β _2_ AR downstream signaling is believed to involve cyclic adenosine monophosphate (cAMP) secondary messenger [10] and induces transcription of choline acetyltransferase (ChAT), an enzyme responsible for ACh synthesis. Functional ChAT expression results in ACh release from T lymphocytes which consequently binds to its receptor α7 nicotinic ACh receptors (α7nAChR) on activated macrophages which downregulates nuclear factor kappa B (NF-κB) dependent pro-inflammatory cytokine production [11]. Thus, spleen is believed to be a central organ for CAP functioning, especially in sepsis and similar inflammatory diseases.

Prostaglandin E_2_ (PGE_2_) is a potent lipid mediator involved in physiological and inflammatory processes and is produced from arachidonic acid by the enzymatic action of cyclooxygenases (COX) (1 and 2) and terminal PGE_2_ synthases such as cytosolic prostaglandin E_2_ synthase (cPGES), microsomal PGES-1 (mPGES-1) and 2. In particular, mPGES-1 is strongly associated with inflammation and displays functional dependency on COX-2 [12]. Among the lipid mediators, special attention has been paid to PGE_2_ which is elevated in chronic inflammatory lesions, as shown by several experimental studies [12]. PGE_2_ levels can increase several folds from picomolar or nanomolar concentrations during inflammation and reach a local concentration of about 10^−6^ M as observed in the rheumatoid joint fluid [13]. The exact biological action of PGE_2_ is constantly debated owing to its contradictory association with pro-inflammatory properties not only in acute inflammation, but also in chronic inflammation *in vivo*, stimulating angiogenesis, inducing pro-inflammatory cytokines and tissue destruction on one hand and inducing strong inhibitory effects on pro-inflammatory cytokines on the other [14, 15].

PGE_2_ is synthesized by most cells and has pleiotropic effects in an autocrine or paracrine manner. In addition, cytokine induced PGE_2_ synthesis and diffusion across the blood brain barrier (BBB) is responsible for sickness behavior, an important consequence of systemic immune response and thus PGE_2_ acts as a principal mediator in bridging the immune and nervous systems [16]. Interestingly, PGE_2_ is linked to the vagal inflammatory reflex at multiple steps. First, animal studies have shown that systemic inflammation increases the discharge activity of afferent vagal sensory nerves in a PGE_2_ dependent manner [17]. Furthermore, EP3 (one of the four PGE_2_ receptors) mediated neural pathways have been implicated in fever induction and hypothalamic-pituitary-adrenal (HPA) axis activation following systemic illness [18]. In addition, activation of EP3 expressing neurons in the vagal nuclei of the brainstem seems to be critical for central autonomic circuits and neural outflow to specific immune organs [19]. In line with this, Macneil et al., have clearly illustrated that central prostaglandin synthesis is essential for the increased activity of the sympathetic splenic nerve following systemic endotoxin administration [20]. There is also accumulating evidence on PGE_2_ involvement in the cholinergic modulation of immune responses in activated cells such as monocytes [21], astrocytes [22] and microglia [23]. Recent studies have given valuable insight into the role played by prostaglandins, whose concentration is increased locally following the activation of CAP, in suppressing the release of TNFα and IL-18 by activated macrophages during endotoxin induced inflammation [21, 24, 25]. These findings are well supported by various *in vitro* studies that have successfully shown PGE_2_ to regulate and suppress TNF production by peripheral blood mononuclear cells [26]. Also, binding of PGE_2_ to its two G-protein coupled receptors EP2 or EP4 has been found to increase intracellular cAMP concentration in various inflammatory cells which is generally known to inhibit effector cell functions [27].

Intriguingly, exogenous treatment of activated T lymphocytes with PGE_2_ mimics the effects of β_2_ AR stimulation by favoring synthesis of Th2 cytokines against Th1 cytokines [10, 25]. Finally, we have recently provided substantial evidence directly linking PGE_2_ to vagus nerve stimulation (VNS) activated CAP functioning where VNS failed to downregulate NF-κB dependent cytokines such as TNFα in endotoxaemic mice lacking mPGES-1 [28]. However, the exact immunological and neural events affected by mPGES-1 deficiency remains to be deciphered.

In the present study, we aimed to dissect the role of mPGES-1 dependent PGE_2_ synthesis in the neuro-immune circuitry of the CAP during endotoxaemia in mice. In particular, we wanted to study the activation of splenic nerve in response to VNS, ACh synthesis and cholinergic immunomodulation in the spleen of mPGES-1 deficient mice both *in vivo* and *in vitro*. In addition, we also aimed to investigate the role of mPGES-1 blockade in the cholinergic modulation of inflammation and immunomodulatory effects of PGE_2_ on lipopolysaccharide (LPS) activated human peripheral blood mononuclear cells (PBMCs) *in vitro*.

## Materials and methods

### Animals

Adult DBA/1lacJ mice (25-30g) with Ptges gene deletion (mPGES-1 -/-) and wildtype congenic controls (mPGES-1 +/+) were produced by in-house breeding of heterozygous or homozygous mice as described previously [29]. Genotypes were confirmed using PCR. All animals were certified to be free from rodent pathogens and were regularly checked for behavior, health and housing by trained animal facility personnel. Mice were housed in a 12 h light/dark cycle at constant room temperature. Food and water were provided *ad libitum*. Mice were allowed to acclimatize for at least one week before they were used for experiments. All procedures were performed according to the guidelines approved by the Regional Ethics Committee (Animal Welfare Committee) at the Karolinska Institutet, Sweden (ethics no N129/13) and all efforts were made to minimize animal suffering.

### Vagus nerve stimulation

All animals were anesthetized using isoflurane and the vagus nerve was isolated under microscopic examination. Briefly, an electrode was placed below the isolated nerve and endotoxin lipopolysaccharide (LPS, L2630 Sigma-Aldrich Sweden AB) (2 mg/kg) was injected intraperitoneally (i.p.). Five minutes later, the vagus nerve was stimulated (5V, 1Hz for 5 min) using an AcqKnowledge software (Biopac Systems, USA) monitored stimulator. The incision was then stitched and animals were allowed to recover in their home cage. Control group were sham operated mice only subjected to superficial neck incision and LPS administration. For measuring NE release in response to VNS, animals were euthanized (CO_2_ inhalation) 30 mins after VNS and spleens were collected in solution containing EDTA (1mM) and sodium metabisulfite (4mM) to prevent catecholamine degradation. Samples were then homogenized using dounce homogenizer, debris was pelleted by centrifugation at 10000 rpm for 10 min, and supernatants were stored at -80°C until further use.

### Cell culture experiments

#### Reagents

Cells were cultured in an assay medium containing RPMI-1640 supplemented with 20% heat inactivated fetal bovine serum (FBS), L-ascorbic acid (75 μM), (+/-)-α-tocopherol (25 μM), 12.5mM D-glucose, streptomycin (100 U/ml) and 1mM sodium pyruvate (all from Sigma-Aldrich, Sweden). Lipopolysaccharide (LPS, L2630), Norepinephrine (A7257), nicotine (N3879) and indomethacin (I7378) were also obtained from Sigma-Aldrich. mPGES-1 inhibitor (compound III), Ficoll-Paque PLUS and 100μM cell strainer (11517532) were purchased from NovaSAID, GE healthcare and Fisher scientific respectively. PGE_2_ (P0409) was obtained from Sigma-Aldrich, dissolved in 99.5% ethanol and further diluted in phosphate buffered saline (PBS) to the working concentration.

#### Primary mouse splenocyte culture and treatment

Animals were euthanized by CO_2_ inhalation and spleens were collected and kept in RPMI medium. Then, a single cell suspension was prepared by passing the spleen through 100μm cell strainer and centrifuged. Later, the cell pellet was resuspended in Ammonium-Chloride-Potassium (ACK) lysis buffer and incubated for 7 min at 37° C to remove red blood cells (RBCs). Following that, cells were counted and approximately 1x10^6^ cells were seeded in 96 well plates. For NE stimulation experiments, cells were first treated with NE (1, 10 or 100 μM) and 30 minutes later, LPS (100ng/ml) was added and incubated for 3 hours. For nicotine experiments, splenocytes were pretreated with nicotine at 100μM for 30 minutes, then stimulated with LPS (10ng/ml) for 3, 6 and 20 hours respectively. In all these experiments, cells were incubated in a humidified incubator with 5% CO_2_ at 37°C for specified hours and cell supernatants were collected, centrifuged and stored at -20°C for cytokine measurement.

#### Primary human PBMC culture

Blood samples were collected from healthy individuals, giving their written consent. Peripheral blood mononuclear cells (PBMC) were isolated from the buffy coats of heparinized blood using Ficoll-Hypaque density centrifugation. In short, the whole blood was dispensed in two 50ml falcon tubes and were filled up to 35ml with 1% PBS. Following this, 12ml of Ficoll-Paque was gently added to the bottom of the tubes respectively. Centrifugation was performed at 1479g for 20 min at 20°C and white buffy layer, thus obtained, was rinsed twice with PBS and cells were finally suspended in 10ml PBS and counted using a hemocytometer. ACK lysis buffer was used to remove RBCs. PBMC, thus prepared, was suspended using RPMI-1640. Cells were seeded at a concentration of 1.5 x 10^6^ cells per ml in a flat-bottomed 96 well plate having a volume of 200 μl per well and incubated at 37°C in a humidified 5% CO_2_ atmosphere. This study was performed according to the guidelines approved by the Regional Ethics Committee at the Karolinska Institutet, Sweden and was conducted according to the principles of the Helsinki Declaration.

#### Treatment of activated human PBMC with nicotine in the presence or absence of mPGES-1 inhibitor

Fresh human PBMC was pretreated with mPGES-1 inhibitor (10 μM) and 30 minutes later, cells were stimulated with LPS (100ng/ml) and nicotine (1, 10 or 100 μM) simultaneously. Since mPGES-1 inhibitor was prepared in dimethyl sulfoxide (DMSO), 0.05% of DMSO was used in the treatment to serve as controls. Cells were incubated for 6, 14 and 20 hours respectively. Cell culture supernatants were collected and stored at -20°C for cytokine analysis.

### Norepinephrine measurement using ELISA

Stored supernatants of spleen extracts were thawed to room temperature and NE content was measured using the Noradrenaline Research competitive ELISA kit (BA E-5200, LDN, Germany). Briefly, NE was extracted using cis-diol-specific affinity gel, acylated and later converted enzymatically. Then, the supernatants are transferred to noradrenaline microtiter plates with precoated antibodies and incubated overnight at 4°C. The analyte competes for specific number of antibody binding sites and unbound analyte is washed away the next day. Following the wash, the antibodies bound to the plate were quantified using anti-rabbit IgG peroxidase conjugate-TMB detection method. The absorbance was read at 450 nm and 630 as reference wavelength. The sensitivity of the kit is 0.1ng/ml * correction factor and each sample were run in duplicates. Each treatment group had 3–4 animals.

### Beta 2 adrenergic receptor expression by flow cytometry

Animals were euthanized by CO_2_ inhalation 30 min after VNS. Freshly extracted spleen was collected in cold FACS buffer (PBS with 1% fetal bovine serum) and a cell suspension was obtained by passing the spleen through a 100μm cell strainer. Red blood cells were lysed using ACK lysis buffer and Fcγ receptors were blocked using monoclonal rat-anti-mouse CD16/CD32 antibodies (14-0161-85, AB_467134, Thermo Fisher, USA) at 1:100 dilution. CD4+ cells were isolated from total splenocytes by positive selection using MACS separation (purity >90%) as per manufacturer’s instructions (CD4 (L3T4) MicroBeads, 130-049-201, Miltenyi biotec, Germany). CD4 enriched cell population was incubated (20 minutes, 4°C, at darkness) with the following panel of antibodies: Rabbit-anti-mouse polyclonal PE ADBR2 (bs-0947R-PE, AB_11113746, Bioss Antibodies, USA) at 1:100 dilution, monoclonal rat-anti-mouse PE/Cy7 CD62L (560516, AB_1645257, BD Biosciences, USA) at 1:200 dilution, monoclonal rat-anti-mouse BV510 CD 4 (100553, AB_2561388, BioLegend, USA) at 1:100 dilution, monoclonal rat-anti-mouse APC Cy7 CD3 (100222, AB_2242784, BioLegend, USA) at 1:200 dilution and monoclonal rat-anti-mouse APC CD44 (103012, AB_312963, BioLegend, USA) at 1:400 dilution. Viability of cells was measures by using LIVE/DEAD^™^ Fixable Green Dead Cell Stain Kit (1:1000) (L23101, ThermoFisher Scientific, USA). Stained cells were fixed using 4% paraformaldehyde (15 minutes, 4°C) and samples were acquired the following day using a BD FACSVerse instrument (BD Biosciences, USA) evaluating a minimum event of 60000 live single cells. Calculation of compensation matrix and analyzation of data was performed using FlowJo v.X software (TreeStar, USA). The compensation was set by using single stained beads and gates were corrected using appropriate fluorescence minus one (FMO) controls. Gating strategy is illustrated in figure A in S1 File.

### Measurement of TNFα using sandwich ELISA

TNFα cytokine levels in cell culture supernatants was quantified using a sandwich DuoSet ELISA kit specific for human (DY210, R&D systems) or mouse (DY410, R&D systems) TNFα accordingly. Assays were performed according to manufacturer’s instructions. Briefly, ELISA microtitre plates were coated with the respective capture antibodies, mouse anti-human TNFα (4μg/ml) and goat anti-mouse TNFα (800ng/ml), incubated overnight at room temperature (100μl/well) and later blocked with the reagent diluent. Samples were assessed using a 7-point standard curve with a high standard of 2000 (mouse) or 1000 (human) pg/ml of the respective recombinant human or mouse TNFα. Samples (100μl/well) were loaded in duplicates and incubated for 2 hours at room temperature. The plates were later incubated with biotinylated goat anti-human or mouse TNFα antibody (100μl/well) for two hours followed by the addition of streptavidin-horse radish peroxidase conjugate (1:200) to each well for 20 min. The plates were washed thrice with 1% PBS containing 0.05% Tween-20 (PBS-Tween) pH 7.2–7.4 after each step. Following the final wash, 100 μl of the substrate solution containing hydrogen peroxide and tetramethylbenzidine in 1:1 ratio was added to the wells. The enzymatic reaction was then terminated after 20 min by the addition of 50 μl of 2N sulphuric acid. The readings were taken at 450nm along with the wavelength correction being set at 562 nm.

### Immunofluorescent staining of Choline acetyltransferase in murine splenocytes

Splenocytes from both mPGES-1 (+/+) and mPGES-1 (-/-) mice were seeded in chamber slides, treated with LPS (10ng/ml) for 20 hours. After LPS treatment, the cells were washed with PBS twice and fixed in 4% paraformaldehyde for 20 minutes. Fixed cells were later used to stain choline acetyltransferase (ChAT) protein using indirect immunofluorescence. Briefly, unspecific binding was blocked by incubating cells with 3% normal mouse and goat serum. Later, primary antibodies against ChAT (polyclonal rabbit-anti-mouse, AB143, AB_2079760, Millipore, USA) at 1:100 dilution, prepared in 3% normal mouse serum, was added to cells and incubated overnight at 4°C. Next day, cells were washed with PBS-tween and incubated with polyclonal goat-anti-rabbit IgG conjugated with Alexafluor 488 (ab150077, AB_2630356, Abcam, UK) at 1:800 dilution for one hour at room temperature. After several washes with PBS, cells were incubated with DAPI for 1 minute and washed again with distilled water. Finally, slides were mounted using PBS-glycerol and stored at 4°C for microscopic analysis. The slides were examined using fluorescence microscope (Leica Microsystems, Cambridge, UK) and images were taken at 40x using Leica application suite version 4.4 software. ChAT positive cells (green) and DAPI positive cells (blue) were counted accordingly in at least 4 consecutive fields and the total number of ChAT^+^ cells was divided by the total number of DAPI ^+^ cells and expressed as % cells expressing ChAT for given number of cells.

### Cytokine profiling of cell culture supernatants using multiplex assay

For our study, a TH1/TH2 human 10-plex assay (K15010C, Human pro-inflammatory panel 1, MSD, USA), ultrasensitive multiplex kit, was purchased from MesoScale Discovery. Using this technique, the concentration of IFNγ, IL-1β, IL-2, IL-4, IL-5, IL-8, IL-10, IL-12p70, IL-13 and TNFα in the culture supernatant was measured. The detection range is 0 to 2500 pg/ml respectively. Each well in the MSD 96-well plate had 10 carbon electrodes, each of which was pre-coated with antibodies against a specific cytokine of interest. Briefly, the wells were blocked with assay diluent (25 μl), sealed and incubated for 30 min at room temperature. Following the addition of samples, standards and controls at 25 μl per well, the plate was sealed and incubated overnight at 4°C with shaking. At the end of the incubation the wells were washed thrice as per the manufacturer’s protocol. After the wash, the detection antibody was added at 25 μl per well to the plate, sealed and incubated for 2h at room temperature. At the end of the incubation, the plate was washed thrice and 150 μl of the MSD Read Buffer was added to each well. The MSD plate was read using the MSD Sector Imager 2400 plate reader. The raw data was measured as electrochemiluminescence signal (light) detected by photo detectors and analyzed using the Discovery Workbench 3.0 software (MSD, USA). A 4-parameter logistic fit curve was generated for each analyte using the standards and the concentration of each sample calculated.

### Statistics

All samples were run in duplicates and data are represented as mean ±SEM from at least 3 independent experiments. Statistical analyses were performed using one-way ANOVA or Student’s T–test. Differences with p value ≤ 0.05 considered as statistically significant. All the statistical tests were done using Prism 6.0 software (Graph Pad, USA).

## Results

### VNS induces splenic norepinephrine release in endotoxaemic mPGES-1 (-/-) mice

**Fig 1:**
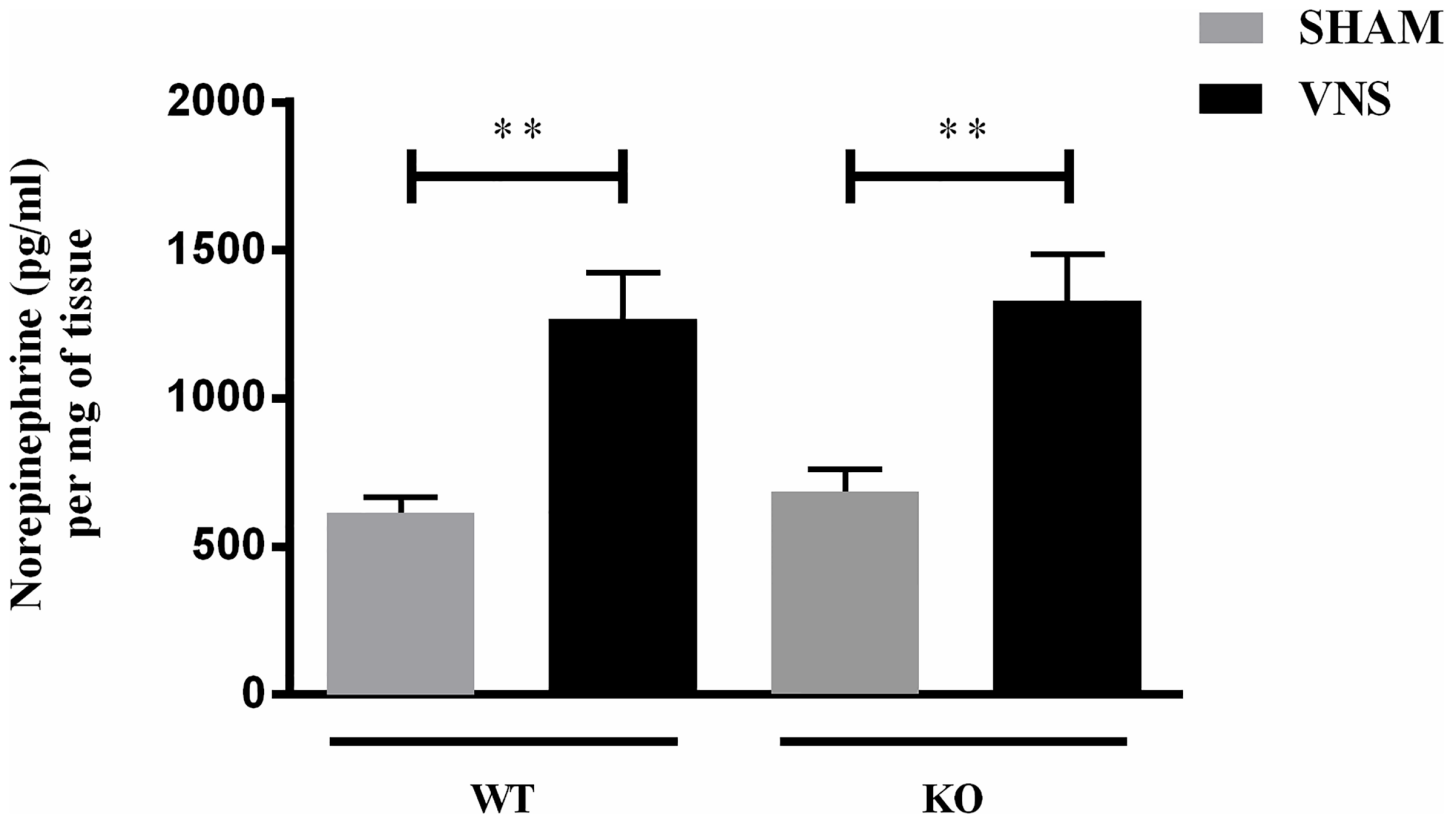
VNS induces splenic norepinephrine release in endotoxaemic mPGES-1 KO mice in vivo. Both DBA/1lacJ m PGES-1 (+/+) (WT) and (-/-) (KO) animals were subjected to either SHAM (n = 4) or VNS (n = 5) after intraperitoneal LPS (2mg/kg) injection. Splenic NE release was quantified 30 minutes after VNS treatment. Each sample was run as duplicates during the assay and values are represented as mean ±SEM from 4–5 individual animals per treatment group (**p<0.01; SHAM vs VNS; One-way ANOVA).

Electrical stimulation of vagus nerve has previously been shown to activate the splenic nerve which results in NE release in mouse spleen [6]. To investigate if mPGES-1 deficiency affects VNS induced splenic NE release *in vivo*, we performed VNS in LPS treated mPGES-1 (+/+) and mPGES-1 (-/-) mice and measured the NE content in the spleen 30 minutes later. As expected, VNS increased the splenic NE production in the LPS treated mPGES-1 (+/+) mice (614.8 ± 52.9 (Sham) vs 1268.2 ±156.7 (VNS); units: pg/ml per mg of tissue; p<0.01). Interestingly, similar upregulation was detected despite mPGES-1 absence in the endotoxaemic mPGES-1 (-/-) mice (685.8 ± 75.2 (Sham) vs 1328.7± 158.4 (VNS); units: pg/ml per mg of tissue; p<0.01) as depicted in Fig 1. This shows that mPGES-1 does not have a significant role in splenic nerve activation following VNS treatment in endotoxaemic mice.

### LPS activated mPGES-1 (-/-) splenocytes display an altered response to NE stimulation

**Fig 2:**
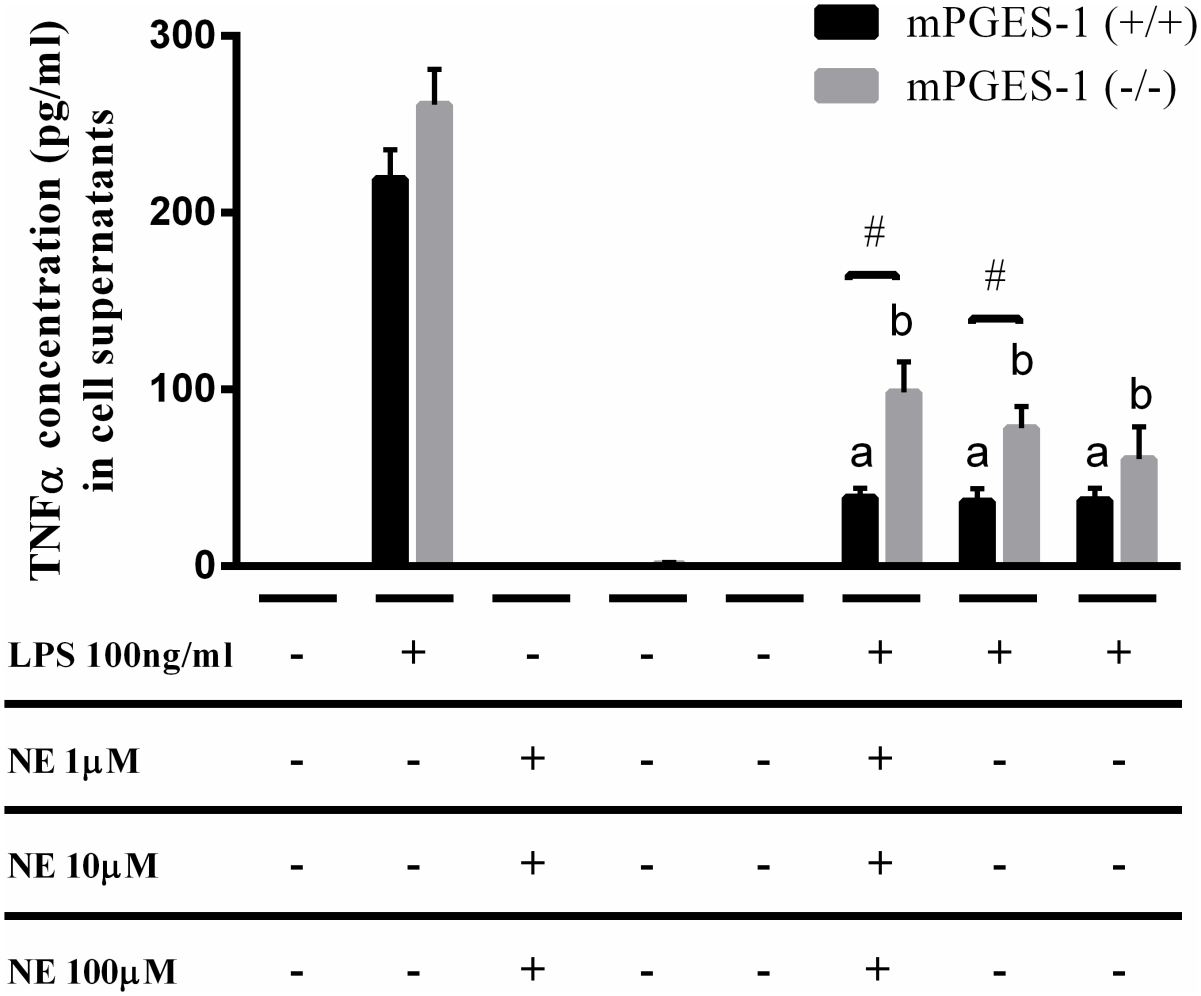
Lipopolysaccharide activated splenocytes lacking mPGES-1 gene expression display an altered response to NE stimulation. Primary splenocyte cultures established from mPGES-1 (+/+) and (-/-) mice were pretreated with norepinephrine (NE) at 1, 10 and 100 μM concentration for 30 mins and then activated with the endotoxin, LPS (100ng/ml). Cell supernatants were analyzed for cytokine production following 3 hours of treatment. ^a,b^ p<0.0001; LPS versus LPS+NE within WT or KO; One-way ANOVA. ^#^ p<0.05; mPGES-1(+/+) versus mPGES-1 (-/-) within LPS+NE treatment; student’s T-test. Each sample was run as duplicates during the assay and values are represented as mean ±SEM from 3 independent experiments.

After confirming that VNS induces NE release in mPGES-1 (-/-) mouse spleen, we next wanted to study the effects of NE on LPS activated mPGES-1 (+/+) vs mPGES-1 (-/-) mouse splenocytes *in vitro*. Both mPGES-1 (+/+) and mPGES-1 (-/-) splenocytes did not produce any detectable levels of TNFα either under basal conditions or in response to NE alone, however LPS treatment strongly increased its production. Interestingly, both mPGES-1 (+/+) and mPGES-1 (-/-) released similar TNFα levels in response to LPS, confirming previous data showing that mPGES-1 (-/-) mice present a normal response to LPS with increase of inflammatory cytokines [30]. NE, irrespective of the dose added, inhibited approximately 82% of the LPS induced TNFα cytokine release from mPGES-1 (+/+) splenocytes (Fig 2). On the contrary, mPGES-1 (-/-) splenocytes displayed a dose dependent TNFα inhibition in response to increasing NE concentration. (62.3% at NE 1μM, 70.1% at NE 10μM,76.9% at NE 100μM and 89.7% at NE 1mM; % inhibition; p<0.0001; n = 3) as shown in Fig 2. In addition, at 1 and 10μM, NE had significantly different levels of TNFα inhibition among the mPGES-1 (+/+) and mPGES-1 (-/-) splenocytes (38.9±5.1 (mPGES-1 (+/+)) vs 98.5±17.2 (mPGES-1 (-/-)); units: pg/ml, p<0.05; n = 3, 1mM NE). At the same time, at higher NE (100μM and 1mM (data not shown)) concentrations, such differences seem to disappear and 82–89% of the LPS induced TNFα production was reduced in both mPGES-1 (+/+) and mPGES-1 (-/-).

**Fig 3:**
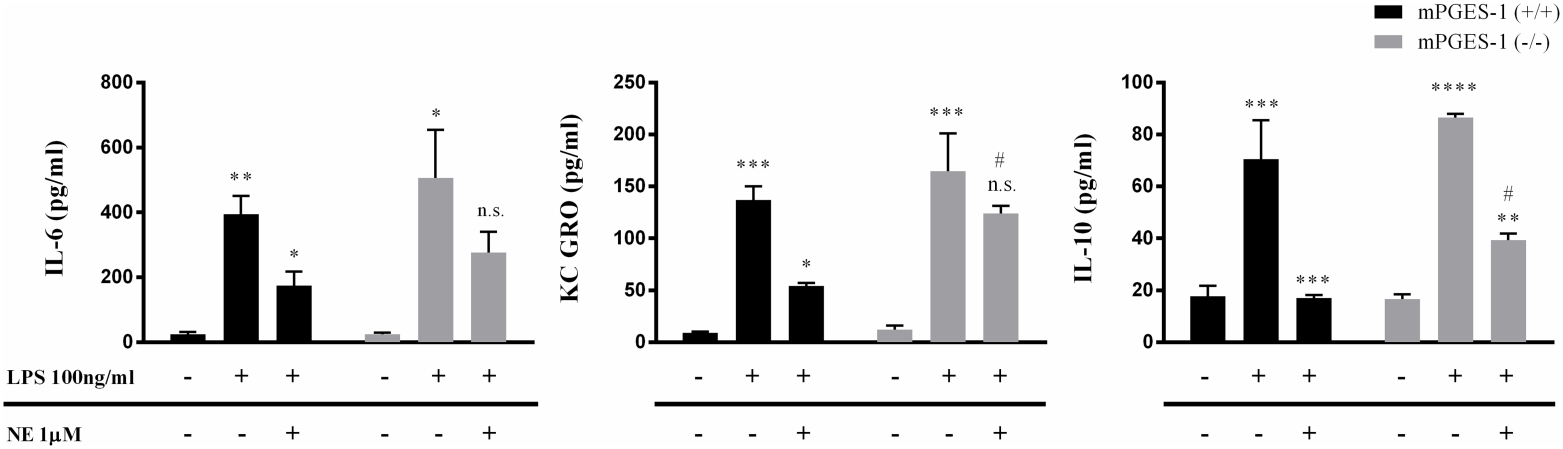
Cytokine profiling of activated mPGES-1 (-/-) splenocytes in response to NE stimulation. Primary splenocyte cultures established from mPGES-1 (+/+) and (-/-) mice were pretreated with norepinephrine (NE) at 1μM concentration for 30 mins and then activated with the endotoxin, LPS (100ng/ml). Cell supernatants were analyzed for cytokine production following 3 hours of treatment. (*p<0.05; LPS versus LPS+NE). (^#^ p<0.05; mPGES-1(+/+) versus (-/-) within LPS+NE treatment; student’s T-test). Each sample was run as duplicates during the assay and values are represented as mean ±SEM from 3 independent experiments. Statistical analysis was done using One-way ANOVA unless otherwise indicated.

Cytokine profiling by multiplex assay confirmed our observations with TNFα (Figure B in S1 File). Similar trends of LPS induced IL-10 increase and subsequent inhibition following NE 1μM was observed in both mPGES-1 (+/+) and mPGES-1 (-/-). Also, NE treatment significantly limited IL-10 release in mPGES-1 (+/+) when compared to mPGES-1 (-/-). While NE treatment strongly reduced LPS induced KC GRO and IL-6 levels, it failed to do so in the absence of mPGES-1 gene expression as depicted in Fig 3. Other cytokines such as IFNγ, IL-2, IL-4, IL-5, IL-1β and IL-12p70 were detected at very low levels (data not shown). Thus, mPGES-1 expression seems to partly control the immunomodulatory effects of NE, especially at lower NE levels.

### mPGES-1 gene deletion does not affect β_2_ adrenergic receptor expression on effector memory T lymphocytes

**Fig 4:**
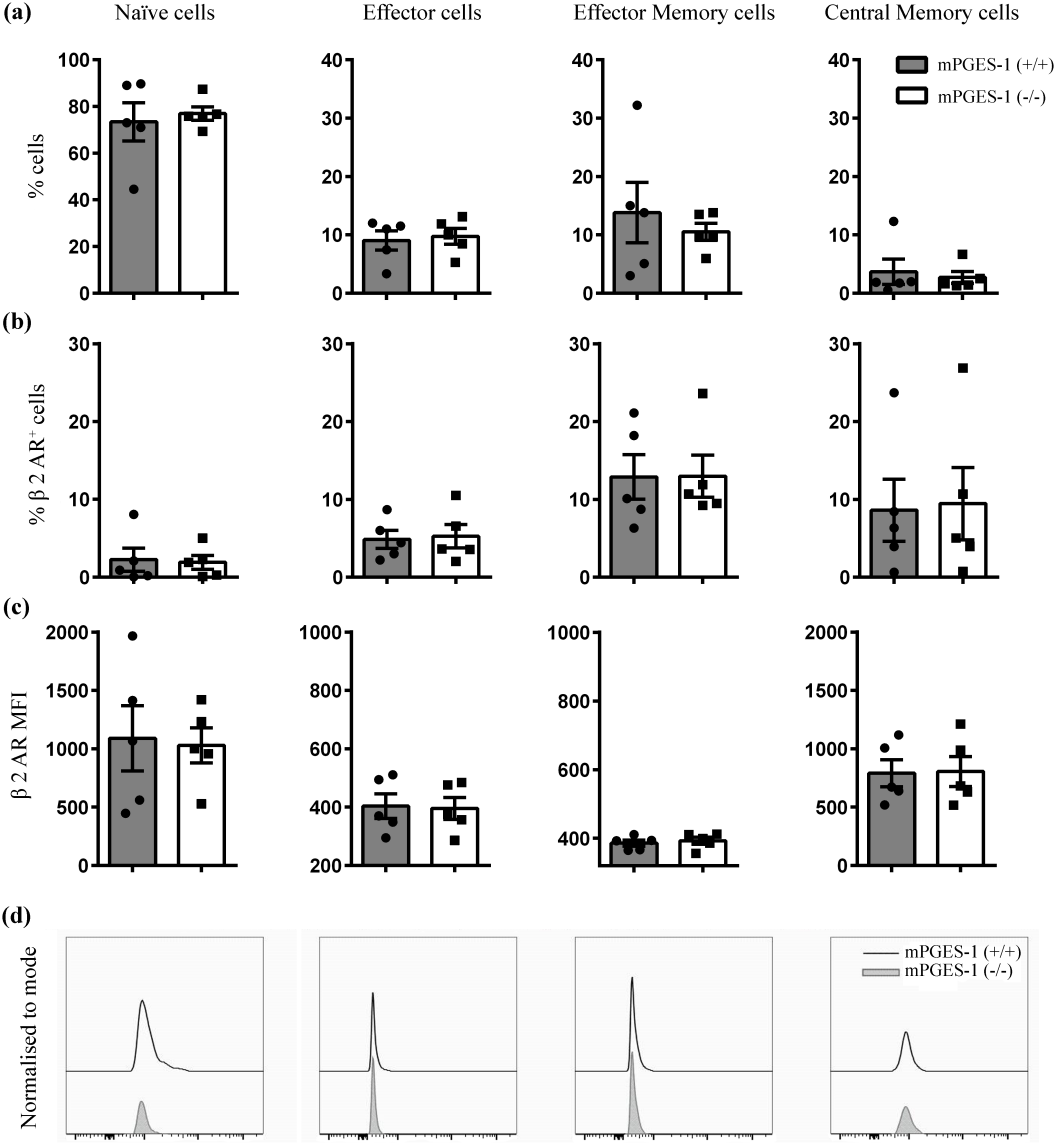
mPGES-1 deletion has no effect on spleen T cell subset relative numbers or their β2 AR expression following VNS in LPS treated mice. Both mPGES-1(+/+) and (-/-) DBA/1lacJ mice were subjected to VNS (n = 5) following intraperitoneal LPS (2mg/kg) injection 30 min before spleen collection. (a) Single cell suspensions of splenocytes were analyzed by flow cytometry in the T cell subsets CD44^lo^ CD62L^hi^ (naïve), CD44^lo^ CD62L^lo^ (effector), CD44^hi^ CD62L^hi^ (central memory) and CD44^hi^ CD62L^lo^ (effector memory) as identified according to figure A in S1 File. The proportions of the different T cell subsets are represented as % of viable CD4+ CD3+ lymphocytes. (b) The relative number of β_2_ AR+ cells among naïve, effector, central memory and effector memory T cell subsets were determined by flow cytometry. The proportions of β_2_ AR+ cells are reported as % of viable T cell subset. (c) β_2_ AR protein levels on the cell surface of naïve, effector, central memory and effector memory T cell subsets were determined by flow cytometry as (geometric) MFI. (d) Representative β_2_ AR histograms. Values are represented as mean ±SEM from 5 individual animals per treatment group.

An integral part of a functioning CAP is ACh production by ChAT+ CD4 memory T cells in response to NE. We therefore investigated β_2_ receptor expression on different subsets of magnetically selected CD4+ T cells (purity >75%) from mPGES-1 (-/-) and (+/+) mouse spleen following VNS. Interestingly, these different T cell subset populations, represented as percentage of CD4+ CD3+ lymphocytes, showed no differences between VNS treated endotoxaemic mPGES-1 (+/+) and (-/-) spleens Fig 4a. Furthermore, β_2_ adrenergic receptor (AR) protein expression on the CD4+ CD44^hi^ CD62L^lo^ effector memory cells in VNS treated spleens, measured both in terms of percentage of CD4+ CD44^hi^ CD62L^lo^ population (12.89±2.9% (mPGES-1 (+/+)) vs 12.98±2.7% (mPGES-1 (-/-)) and mean fluorescence intensity (MFI) (386±8.8 (mPGES-1(+/+)) vs 392.8±10.3 (mPGES-1 (-/-)), showed no striking differences between mPGES-1 (+/+) and (-/-) as shown in Fig 4b and 4c, with representative β_2_ AR MFI histograms in Fig 4d. We also observed similar levels of β_2_ AR expression among the other T cells subsets despite mPGES-1 gene deletion.

### LPS fails to increase choline acetyltransferase expression in mPGES-1 (-/-) splenocytes

**Fig 5:**
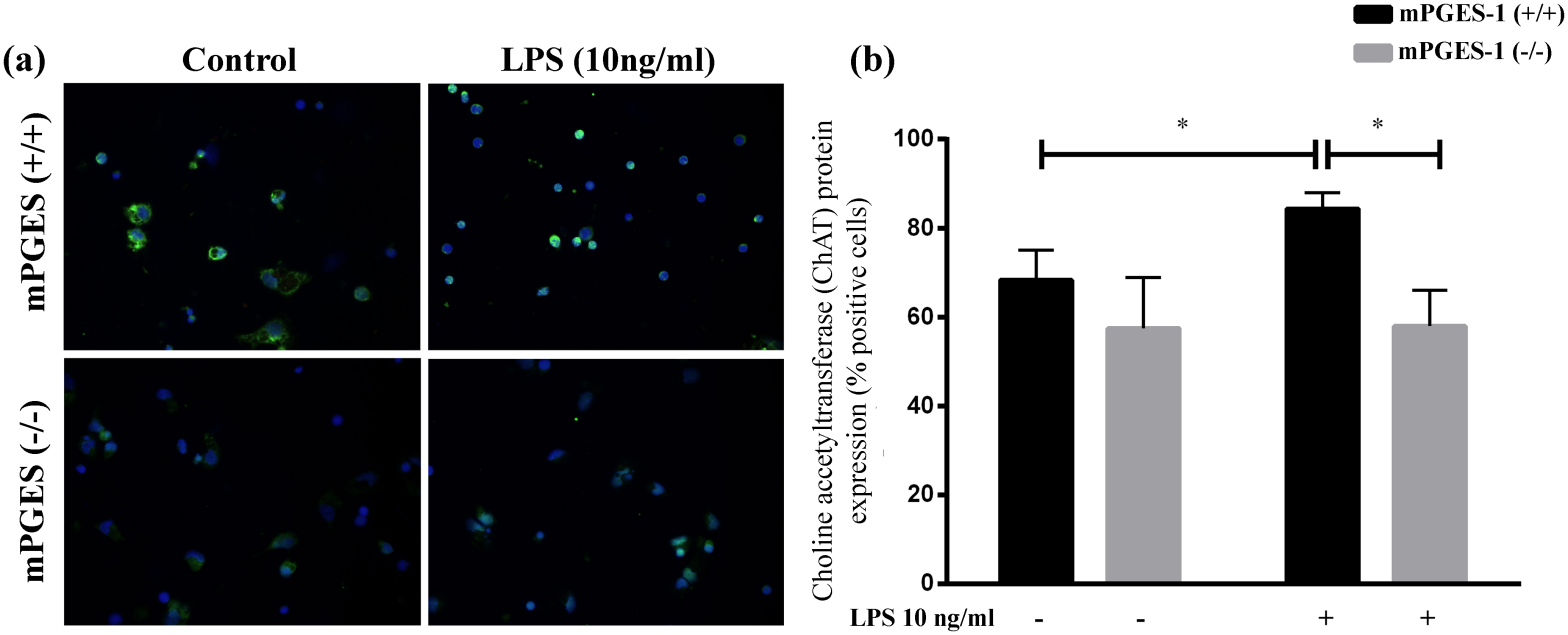
LPS fails to increase choline acetyltransferase (ChAT) expression in the absence of mPGES-1 expression. Primary splenocyte cultures from mPGES-1(+/+) and (-/-) mice were grown on chamber slides and treated with the endotoxin, LPS (10ng/ml). Following 20 hour treatment, cells were paraformaldehyde fixed and stained for ChAT protein expression. (a) Representative microscope images showing ChAT staining (green) and nuclear staining with DAPI (blue). (b) Microscopic analysis of unstimulated and LPS treated mPGES-1(+/+) and (-/-) splenocytes. (* p<0.05; LPS versus control within mPGES-1(+/+); Mann-Whitney Test). (*p<0.05; mPGES-1(+/+) versus (-/-) within the LPS treatment group; Mann-Whitney Test). Each treatment condition was performed in duplicates on the chamber slides and values are represented as mean ±SEM. Quantitative analysis for ChAT positive cells includes measurement of 7 different fields obtained from two independent experiments.

Another critical step in the CAP is the ACh production in the mouse spleen following splenic nerve activation. We have previously shown that mPGES-1 (-/-) mice have defective splenic ACh release following VNS [28]. Here, we measured the ChAT protein expression in LPS stimulated mouse splenocytes and compared it with that from mPGES-1 (-/-) mice *in vitro* as illustrated in Fig 5a. Unstimulated splenocytes from both mPGES-1 (+/+) and mPGES-1 (-/-) expressed comparable ChAT protein levels (68.4±6.8 (mPGES-1 (+/+)) vs 57.5±11.5 (mPGES-1 (-/-)); units: % ChAT positive cells; n.s.). Moreover, LPS treatment caused a significant increase in ChAT expression of mPGES-1 (+/+) splenocytes (68.4±6.8 vs 84.3±3.6; units: % ChAT positive cells; p<0.05). Intriguingly, mPGES-1 deficient splenocytes failed to display such an increase in ChAT expression in response to LPS (57.5±11.5 vs 57.9±8.1; units: % ChAT positive cells; n.s.) as seen in Fig 5b. These results reiterate our finding that mPGES-1 seems to play an important role in ACh production in response to VNS, and might be crucial in LPS-induced ChAT expression.

### mPGES-1 gene deletion reverses nicotine immunomodulatory effects on LPS activated mouse splenocytes

**Fig 6:**
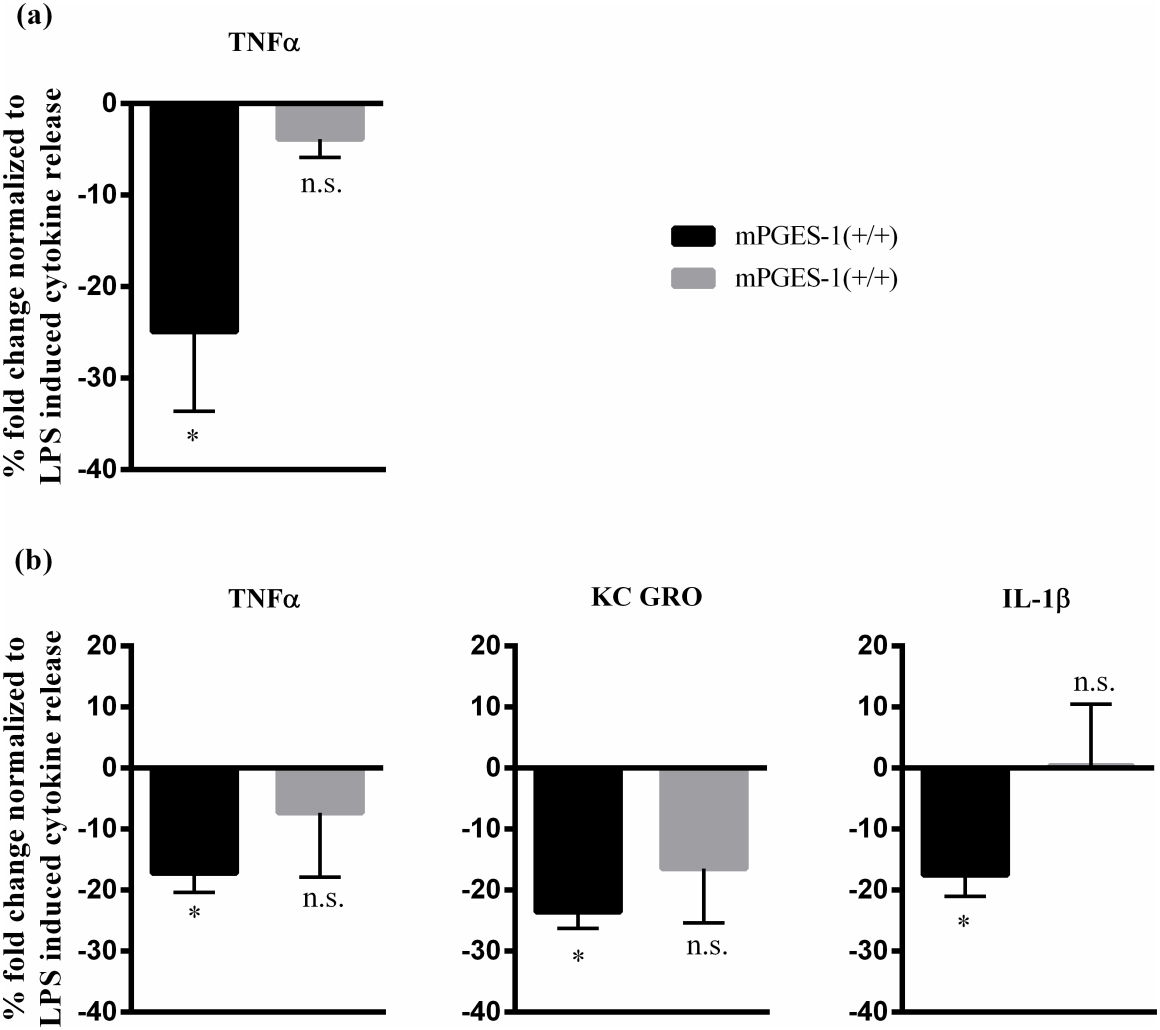
Immunomodulatory effects of α7nAChR agonist nicotine on LPS activated mouse splenocytes is reversed by mPGES-1 gene deletion. Primary splenocyte cultures of mPGES-1(+/+) and (-/-) mice were pretreated with nicotine (100 μM) for 30 mins and later activated with the endotoxin, LPS (10ng/ml). Cell supernatants were analyzed for LPS induced cytokine production following 3 hour incubation. (a) TNFα as measured in cell culture supernatants by ELISA. (*p<0.05; LPS versus LPS+Nicotine within WT; One-way ANOVA, n.s. p>0.05; WT versus KO within LPS+Nicotine treatment group; One-way ANOVA). (b) Fold change of TNFα, KC Gro and IL-1β as measured in cell culture supernatants by Multiplex assay (*p<0.05; LPS versus LPS+Nicotine within WT; One-way ANOVA). Each sample was run as duplicates during ELISA and values are represented as mean ±SEM from 3 independent experiments. Due to high variations between individual experiments, cytokine production in each group was normalized to TNF α level induced by LPS and represented as % fold change.

Next, we investigated if exogenous treatment of activated mPGES-1 (-/-) splenocytes with a potent α7nAChR agonist, nicotine, can mimic the inhibitory effects of VNS on TNFα cytokine production *in vitro*. Both mPGES-1 (+/+) and mPGES-1 (-/-) splenocytes were stimulated with LPS alone or in combination with nicotine at 100μM. In line with literature [30], LPS increased TNFα production multi-fold irrespective of mPGES-1 expression. While nicotine treatment (3 hours) strongly inhibited LPS induced TNF α production in mPGES-1 (+/+), such a limiting response was reversed in the absence of mPGES-1 expression (-24.9±8.7 (mPGES-1 (+/+)) vs -3.9±1.9 (mPGES-1 (-/-)); units: % fold change normalized to LPS treatment alone (100%); p<0.05; n = 4) as displayed in Fig 6a. In addition, nicotine treatment also limited KC GRO and IL-1β release from LPS activated mPGES-1 (+/+) splenocytes (-23.6±2.7 & -17.6±3.5 respectively; units: % fold change normalized to LPS treatment alone (100%); p<0.05; n = 3), however such effects were negated by mPGES-1 gene deletion as depicted in Fig 6b. Other cytokines were detected at very low levels (data not shown). Thus, despite its availability in the milieu, nicotine failed to replicate its inhibitory effects on LPS activated splenocytes lacking mPGES-1 expression. This illustrates that functional mPGES-1 protein is essential for nicotine downstream effects in activated immune cells.

### Pharmacological blockade of mPGES-1 impairs nicotine effects in human PBMC

**Fig 7:**
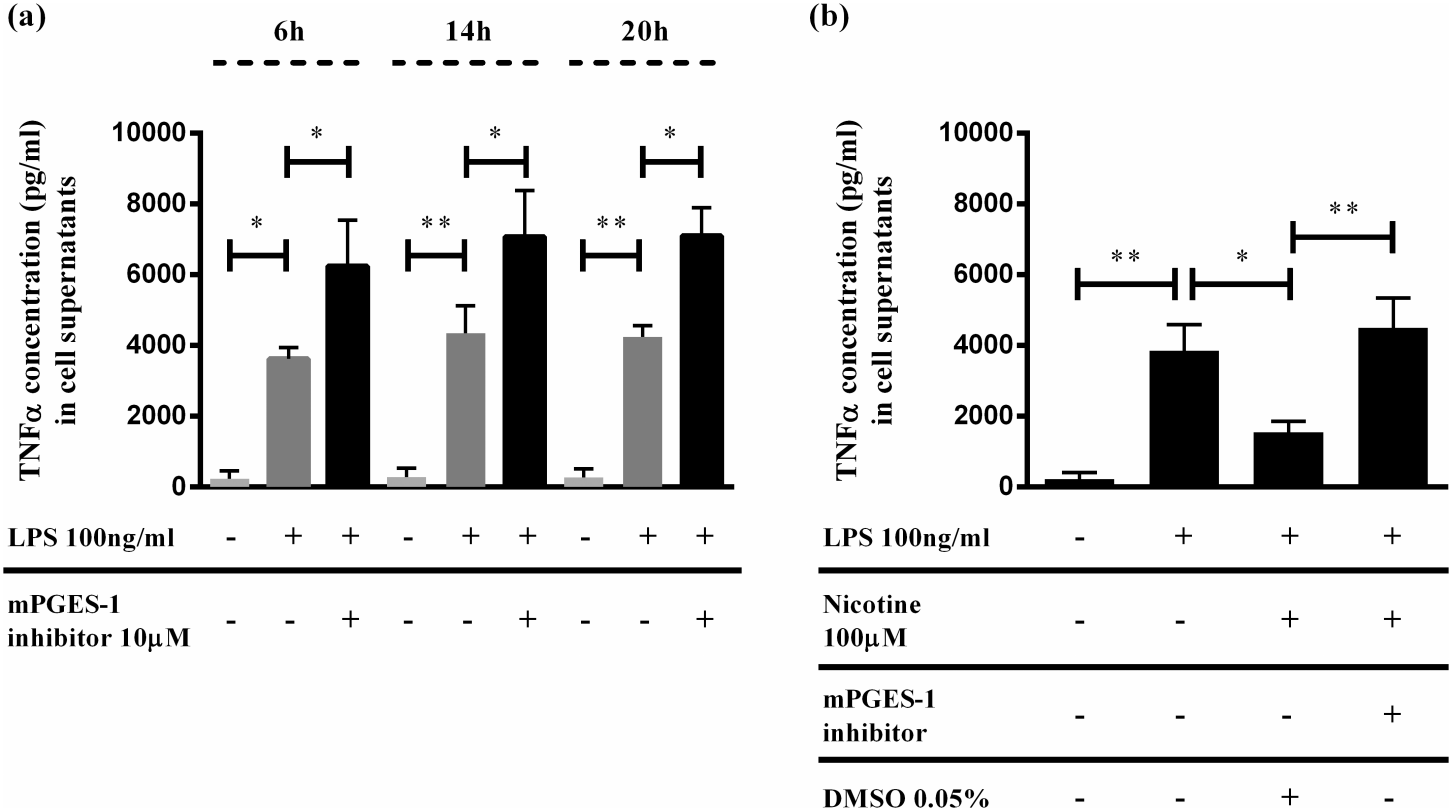
*In vitro* mPGES-1 blockade impairs nicotine’s limiting effects on TNFα production by LPS activated human peripheral blood mononuclear cells (PBMCs). Human PBMC cultures were freshly prepared from healthy blood donors using ficoll density gradient separation. (a) TNFα levels measured by ELISA in culture supernatants after treatment with endotoxin, LPS (100ng/ml) for 6, 14 and 20 hours respectively. Untreated cells served as control. TNFα levels in the culture supernatants were measured by ELISA (*p>0.05, **p<0.01; control versus LPS; One-way ANOVA, *p>0.05; LPS versus LPS+mPGES-1 inhibitor; One-way ANOVA). (b) TNFα levels measured by ELISA in culture supernatants after treatment with nicotine (100 μM) for 14 hours. (*p>0.05; LPS versus LPS+ Nicotine; One-way ANOVA, **p>0.05; LPS+ Nicotine versus LPS+Nicotine+mPGES-1 inhibitor; One-way ANOVA). Each sample was run as duplicates during ELISA and values are represented as mean ±SEM from (a) 3 and (b) 4 independent experiments.

To study the role of mPGES-1 in LPS induced cytokine release and cholinergic modulation of acute immune response in the human setting, we stimulated freshly prepared human PBMC with LPS (100ng/ml) for 6, 14 and 20 hours respectively. Treatment with LPS initiated strong TNFα release into the supernatants at 6 hours and increased further at 14 hours and remained constant till 20 hours. Addition of selective mPGES-1 inhibitor (compound III) [31] resulted in a strong upregulation of TNFα levels compared to LPS alone at all time points as depicted in Fig 7a. Cholinergic modulation of LPS induced immune response was investigated by incubating activated human PBMC with nicotine which led to a significant inhibition of TNFα synthesis and this effect was strongly negated by mPGES-1 functional blockade as shown in Fig 7b. A similar trend was also observed at 20 hours (data not shown). These results clearly highlight the immunomodulatory role of mPGES-1 dependent PGE_2_ synthesis during the cholinergic regulation of acute inflammation.

## Discussion

**Fig 8:**
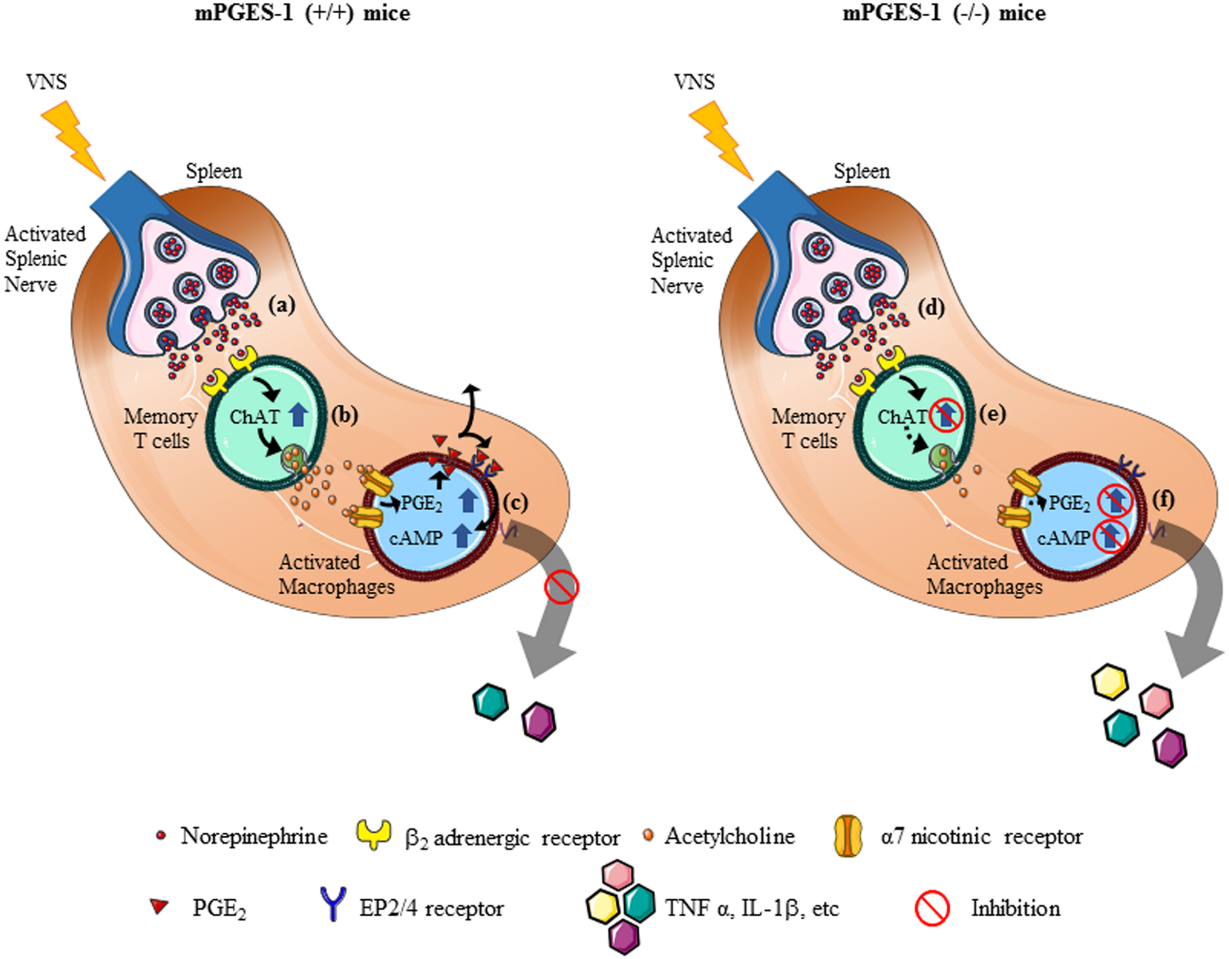
Schematic representation of neuro-immune circuit in the mouse spleen in response to VNS. In an endotoxaemic mPGES-1(+/+) mouse model, VNS induce norepinephrine (NE) release from the splenic nerve which is in close proximity to the CD4^+^ CD44^hi^ Cd62L^lo^ T cells (CD4^+^ memory T cells) (a). NE, thus released, binds to its β_2_ adrenergic receptor (AR) and stimulates choline acetyltransferase (ChAT) expression in the effector cell (CD4^+^ memory T cells) leading to release acetylcholine (ACh) (b). ACh- α7 nicotinic receptor interaction on macrophages increases endogenous PGE_2_ synthesis. Subsequent activation of PGE_2_ receptors EP2/4 causes cAMP upregulation and inhibits cytokine release, thereby controlling inflammation (c). In our current study, we illustrate that in mice with mPGES-1 genetic deletion (mPGES-1 (-/-)), VNS induced sympathetic SN NE release (d) and activation and β_2_AR expression on effector memory cells is intact (e). However, other VNS related molecular events such as choline acetyltransferase (ChAT) dependent ACh release (e) and inhibition of cytokines in response to nicotine (α7nAChR agonist) are impaired (f), thereby clearly demonstrating the role of mPGES-1 dependent PGE_2_ as a crucial mediator in the cholinergic processes related to VNS.

The idea of activating CAP by electrical stimulation of vagus nerve to control acute and chronic inflammation is gaining momentum as a potential therapeutic strategy to treat inflammatory diseases. Our earlier studies in mPGES-1 (-/-) mice have shown that PGE_2_ may constitute an important regulator of the optimal functioning of VNS [28] and ACh release in response to VNS was deficient in mPGES-1 (-/-) mice [28]. Here, we show that mPGES-1 dependent PGE_2_ synthesis is not mandatory for VNS induced splenic NE release and that β_2_ AR expression on CD4^+^ CD44^hi^ CD62L^lo^ lymphocytes is not affected in mPGES-1 (-/-) mice. However, mPGES-1 gene depletion leads to altered capability of NE to inhibit TNFα release by LPS activated splenocytes *in vitro*. In support of our previous findings, LPS failed to increase ChAT protein levels in mPGES-1 (-/-) splenocytes when compared to mPGES-1 (+/+) splenocytes. Interestingly, mPGES-1 gene deletion also reversed cholinergic immunomodulatory effects on activated mouse splenocytes. Similar results were obtained in case of LPS stimulated human PBMCs where the limiting effect of nicotine on TNFα production was reversed following mPGES-1 inhibition. In summary, as illustrated in Fig 8, we report for the first time that central mPGES-1 dependent PGE_2_ synthesis does not play a vital role in VNS related splenic NE release. However, we observed a strong interdependency between mPGES-1 dependent PGE_2_ and cholinergic immunomodulation of activated immune cells which reinstates PGE_2_ to be a part of yet to be discovered regulating system of CAP.

Intravenous LPS administration in animal models has previously been shown to increase the sympathetic outflow to the spleen. Furthermore, while blockade of central prostaglandin synthesis by intracerebroventricular (i.c.v) administration of indomethacin in endotoxaemic rat models impaired sympathetic nerve activity in the spleen, i.c.v treatment with PGE_2_ alone increased splenic nerve activity [20]. It is also important to note that neither LPS injected directly into the central nervous system (CNS) nor PGE_2_ administered peripherally elicit any increase in the sympathetic outflow in the spleen [20]. It is, however, still not studied if VNS related splenic nerve activity is also dependent on PGE_2_ in general. In our earlier studies, we have confirmed that mPGES-1 deletion strongly and specifically affected PGE_2_ synthesis, both in the brain and spleen and we did not detect any shunting towards other prostaglandins in the spleen. We mainly focused on NE release in the spleen post VNS based on the observation of Vida et al., that VNS significantly increases only NE levels and does not affect epinephrine production in mice [8]. From our current *in vivo* experiments, we infer that, despite mPGES-1 gene deletion, VNS induced splenic NE release to a similar extent in both wildtype and mPGES-1 (-/-) mice, which overrules any possible involvement of central nervous PGE_2_ in sympathetic splenic nerve regulation of immune mechanisms in this context.

After ruling out the possibility that impaired splenic nerve activation might be the reason behind improper functioning of VNS in mPGES-1 (-/-) mice, we decided to investigate the key cellular and molecular events following VNS in the isolated splenocytes from mPGES-1 (+/+) and mPGES-1 (-/-) mice *in vitro*. These mechanisms include (i) ability of NE to inhibit LPS induced cytokine release, (ii) ChAT expression following LPS treatment and (iii) α7nAChR activation resulting in TNFα inhibition. Usually, splenocytes prepared from unimmunized mice are mainly composed of ~ 40% T (CD4^+^) and 40% B (B220^+^) lymphocytes, 4% macrophages (CD11b^+^ F4/80^+^), 0.5% dendritic cells (CD11b^+^ CD11c^+^) and 3.2% neutrophils (CD11b^+^ Gr-1^+^) of the total cell population [32]. Though lymphocytes are in majority, splenic macrophages are the major source of TNFα and other pro-inflammatory cytokines during LPS challenge. Thus, Rosas-Ballina et al., have advocated macrophages in red pulp and marginal zone of spleen to be key targets of CAP in bringing down inflammation. Moreover, these LPS activated macrophages is located in juxtaposition to the T lymphocyte—catecholaminergic nerve endings synapse that are activated in response to VNS. [33]

In 2011, Vida et al clearly demonstrated the anti-inflammatory effects of NE (50μM) on LPS activated mouse splenocytes *in vitro*. By the use of classical α and β AR antagonists and splenocytes from β_2_ AR (-/-) mice, they also proved that such effects of NE were strictly executed through β_2_ AR stimulation. Binding assays have estimated between 200–750, 200–600 and 2700–5000 βAR binding sites per T, B and macrophage cell respectively [10, 34]. In agreement with this, we found a significant inhibition of TNFα production by LPS activated mPGES-1 (+/+) and mPGES-1 (-/-) splenocytes in response to NE. However, at most widely used concentrations of 1 and 10 μM, the percentage of NE-mediated TNFα inhibition was lower in mPGES-1 (-/-) splenocytes than in mPGES-1 (+/+). Altogether, this suggests that anti-inflammatory effects of NE might be partially dependent on mPGES-1 induced PGE_2_ release. Intriguingly, splenic nerve stimulation in α7nAChR (-/-) mice suppressed serum TNFα levels and this observation confirms the probable direct modulatory effects of NE on innate immune cells via β_2_ AR signaling [6]. Similar results of NE-β_2_ AR interaction on macrophages and other immune cells strongly limiting LPS induced pro-inflammatory cytokine release have been documented [35, 36]. More in detail, β_2_ AR activation has been shown to protect LPS activated macrophages through enhancement of anti-inflammatory M2 macrophage phenotype [37, 38]. In our *in vitro* experiments, we could confirm NE-induced down regulation of pro-inflammatory cytokines, such as IL-1β, TNFα and KC GRO. Also, IL-10 was hampered, which may be interpreted that NE had a pronounced suppressing effect on macrophages, usually the major IL-10 producer. We also found that IL-1β and KC GRO release from mPGES-1 deficient splenocytes were not affected by NE stimulation, which is in line with PGE_2_ involvement in the downstream effects of β_2_ AR activation. Interestingly, Mackenzie et al., have discovered similar molecular effects dependent on cAMP-PKA pathway to be responsible for the inhibitory effects of PGE_2_ treatment on inflammation [39]. Whether NE attenuates LPS induced TNFα levels by its direct effects on splenic macrophages, or indirectly through T-cell acetylcholine synthesis, is not elucidated. To support the latter, detailed studies *in vivo* have established that β_2_ ARs on CD4+ lymphocytes [8] and CD44^hi^ CD62L^lo^ in particular [9], a subpopulation of memory cells, are critical for VNS to control inflammation during endotoxaemia. Interestingly, in the present study, gene deletion of mPGES-1 affected neither the different T cell subsets in general nor β_2_ AR expression on CD4+ CD44^hi^ CD62L^lo^ effector memory cells in VNS treated endotoxaemic mouse spleen. However, these data do not exclude a potential impact of PGE_2_ on β_2_AR function, which still remains elusive.

Several studies have illustrated the ability of non-neuronal cells to actively secrete ACh upon activation through ChAT enzymatic action on choline and this molecular event is indispensable for the functioning of VNS. In 2004, Suenaga et al., showed that EP4 activation on activated human leukemic T cells (MOLT-3) led to increase in ChAT transcription and ACh production [40]. In line with previous studies on ChAT expression in mouse splenocytes [41], we demonstrated that the induction with LPS for 20 hours resulted in an increase in cytosolic ChAT protein expression. Furthermore, inability of LPS to increase ChAT expression in mPGES-1 (-/-) splenocytes provides further explanation for the defective ACh synthesis in response to VNS in mPGES-1 deficient mice. In addition, with respect to the choline availability for ACh production, *in vivo* studies in LPS treated mice showed that VNS does not increase the choline content in mPGES-1 (+/+) spleen and importantly, mPGES-1 deficiency does not affect the choline production in mPGES-1 (-/-) mice (n = 5 per group, as shown in figure C in S1 File).

Nicotine (a potent α7nAChR agonist) immunomodulatory effects on LPS activated immune cells, both in the periphery (macrophages) and CNS (microglia and astrocytes) are well established [11, 22, 42]. Numerous studies have demonstrated that α7nAChR activation in above mentioned cells led to COX-2 dependent PGE_2_ synthesis which in turn increased cAMP levels intracellularly, thereby inhibiting NF-κB and subsequent cytokine production [21, 25, 43]. In fact, PGE_2_ is known to exhibit its macrophage deactivating properties by strong inhibition of TNF-α production accompanied by a significant increase in IL-10 secretion (16, 24). In corroboration with literature, blockade of PGE_2_ synthesis by mPGES-1 gene deletion reversed nicotine inhibitory effects on LPS induced mouse splenocytes. Thus, even if sufficient levels of ACh or its agonist are administered to mPGES-1 (-/-) mice, their ability to control inflammation caused by activated macrophages might be disrupted owing to low levels of PGE_2_. Furthermore, parallel experiments carried out for translational purposes in LPS activated human PBMC confirmed our earlier observations where mPGES-1 blockade with the selective inhibitor (compound III) resulted in enhanced TNF-α synthesis and in addition, impeded nicotine inhibitory effects. Based on our current findings, we want to go a step further and suggest that mPGES-1, working downstream of COX-2, might be an important enzyme in PGE_2_ production following cholinergic treatment.

### Conclusions

Promising results from the clinical trials of VNS treatment in rheumatoid arthritis (RA) patients [44] is looked upon as a big achievement in the development of bioelectric medicine for treating inflammatory diseases. Therapeutic application of VNS is not only restricted to RA but various studies have shown beneficial effects in Crohn’s disease [45], obesity [46], type 2 diabetes [47], myocardial infarction[48], Alzheimer’s [49], stroke recovery [50] and many others. It is, thus, of paramount importance that the mechanism of action of VNS is dissected in great detail to enhance its efficacy and avoid potential side effects. While various mPGES-1 inhibitors are now being developed for controlling inflammation [12], here we hint at the possible role of mPGES-1 dependent PGE_2_ as a crucial mediator in VNS anti-inflammatory effects. We believe that our study will form the basis and be pivotal in initiating more experimental and clinical studies in this line of research to better understand the vagal inflammatory reflex.

## Supporting information

S1 File Supplementary figures A-C. (PDF)

S2 File Experimental data. (XLS)
