# Correction: Microsomal prostaglandin E synthase-1 gene deletion impairs neuro-immune circuitry of the cholinergic anti-inflammatory pathway in endotoxaemic mouse spleen

**DOI:** 10.1371/journal.pone.0196806

**Published:** 2018-04-26

**Authors:** Priya Revathikumar, Johanna Estelius, Utsa Karmakar, Erwan Le Maître, Marina Korotkova, Per-Johan Jakobsson, Jon Lampa

Fig C is missing from S1 File. The updated S1 File can be viewed below.

## Supporting information

S1 File Supplementary figures A-C. (PDF)
