# Supplementary material for: Microsomal prostaglandin E synthase-1 gene deletion impairs neuro-immune circuitry of the cholinergic anti-inflammatory pathway in endotoxaemic mouse spleen
Source: PLoS One. 2018 Feb 22;13(2):e0193210. doi: 10.1371/journal.pone.0193210 (PMC5823444; doi:10.1371/journal.pone.0193210)
Supplement: S1 File — (PDF) [file pone.0193210.s001.pdf]

Supplementary Figures

S1 Fig.

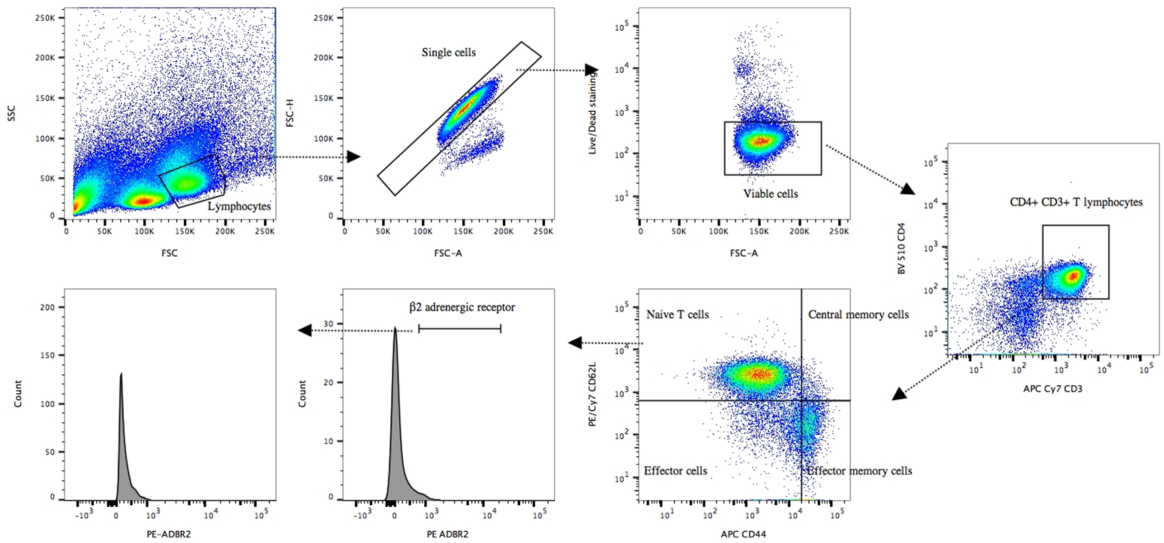

S1 Fig. Gating strategy for $CD4^+$ $CD3^+$ T lymphocytes and their different subsets.

Lymphocytes were gated using SSC-A and FSC characteristics followed by stepwise exclusion of non-single cells and non-viable cells. In viable, single CD4 T lymphocytes ($CD4^+$ $CD3^+$) CD44 and CD62L were used to distinguish between different CD4+ T cell subsets. Corresponding β_2 expression on these subsets were measured as % β_2^+ cells of parent population or mean fluorescence intensity using histograms.

S2 Fig.

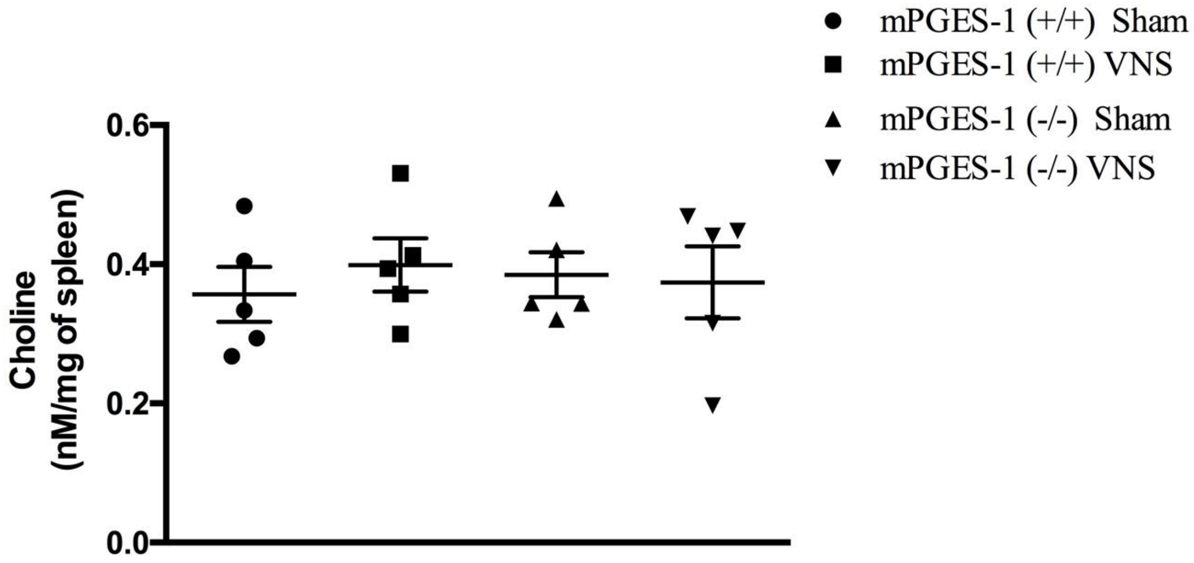

S2 Fig. Choline measurement in mouse spleen following SHAM and VNS treatment. mPGES-1 (+/+) and (-/-) mice underwent SHAM and VNS for 30 minutes and spleens were homogenized for choline measurement. No differences were observed between both treatment groups and mPGES-1 depletion does not affect the choline levels. Samples were run as duplicates during the choline assay and values are represented as mean ±SEM from 5 individual animals per treatment group $p>0.05$; One-way ANOVA)

S3 Fig.
