# Supplementary material for: Correction: Microsomal prostaglandin E synthase-1 gene deletion impairs neuro-immune circuitry of the cholinergic anti-inflammatory pathway in endotoxaemic mouse spleen
Source: PLoS One. 2018 Apr 26;13(4):e0196806. doi: 10.1371/journal.pone.0196806 (PMC5919627; doi:10.1371/journal.pone.0196806)
Supplement: S1 File — (PDF) [file pone.0196806.s001.pdf]

# Supplementary Figures

## Figure A.

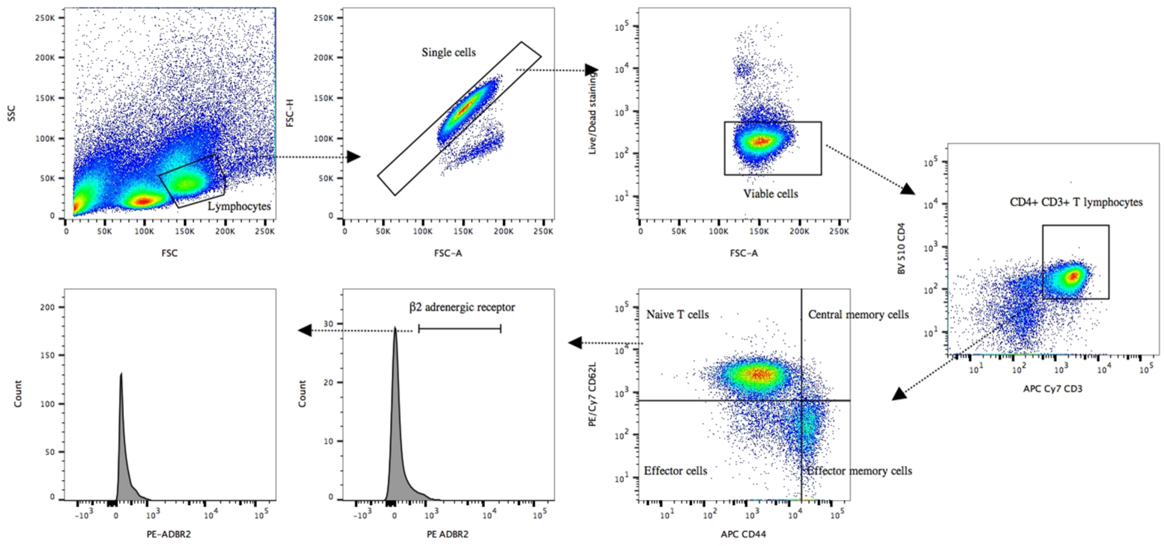


**Fig A. Gating strategy for CD4⁺ CD3⁺ T lymphocytes and their different subsets.**

Lymphocytes were gated using SSC-A and FSC characteristics followed by stepwise exclusion of non-single cells and non-viable cells. In viable, single CD4 T lymphocytes ($CD4^+$ $CD3^+$) CD44 and CD62L were used to distinguish between different CD4+ T cell subsets. Corresponding $\beta_2$ expression on these subsets were measured as % $\beta_2^+$ cells of parent population or mean fluorescence intensity using histograms.

**Figure B**

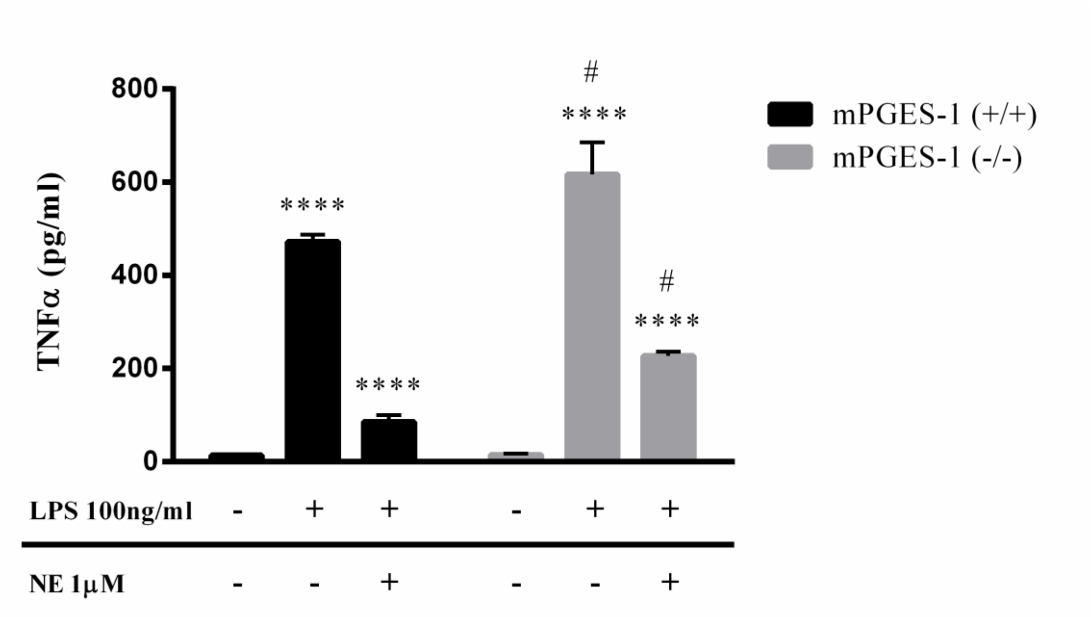


**Fig B. Cytokine profiling of activated mPGES-1 (-/-) splenocytes in response to NE stimulation.** Primary splenocyte cultures established from mPGES-1 (+/+) and (-/-) mice were pre-treated with norepinephrine (NE) at 1μM concentration for 30 mins followed by LPS (100ng/ml) activation. Cell supernatants were analyzed for cytokine production following 3 hours of treatment. (****p<0.0001; LPS versus LPS+NE). ($^{\#}$ p<0.05; mPGES-1(+/+) versus (-/-) within LPS+NE treatment; student's T-test). Each sample was run as duplicates during the assay and values are represented as mean ±SEM from 3 independent experiments. Statistical analysis was done using One-way ANOVA unless otherwise indicated.

**Figure C.**

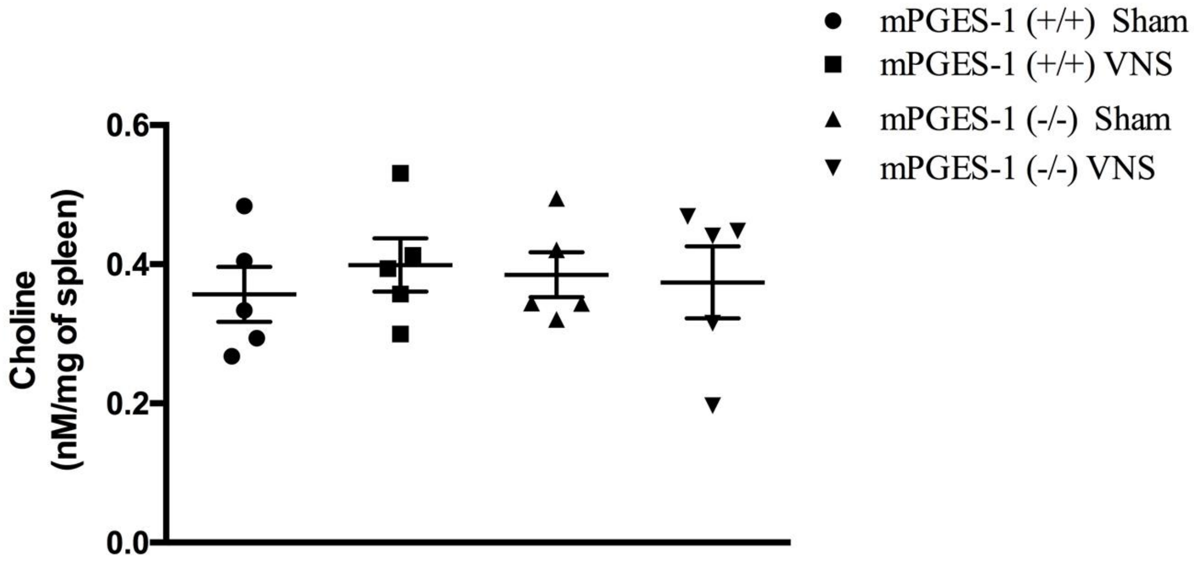


**Fig C. Choline measurement in mouse spleen following SHAM and VNS treatment.** mPGES-1 (+/+) and (-/-) mice underwent SHAM and VNS for 30 minutes and spleens were homogenized for choline measurement. No differences were observed between both treatment groups and mPGES-1 depletion does not affect the choline levels. Samples were run as duplicates during the choline assay and values are represented as mean ±SEM from 5 individual animals per treatment group $p>0.05$; One-way ANOVA)
